# Low intensity repetitive transcranial magnetic stimulation modulates skilled motor learning in adult mice

**DOI:** 10.1038/s41598-018-22385-8

**Published:** 2018-03-05

**Authors:** Alexander D. Tang, William Bennett, Claire Hadrill, Jessica Collins, Barbora Fulopova, Karen Wills, Aidan Bindoff, Rohan Puri, Michael I. Garry, Mark R. Hinder, Jeffery J. Summers, Jennifer Rodger, Alison J. Canty

**Affiliations:** 1 Experimental and Regenerative Neurosciences, School of Animal Biology, University of Western Australia, Perth, Australia; 2 Neuronal Rhythms in Movement Unit, Okinawa Institute of Science and Technology Graduate University, Okinawa, Japan; 3 Wicking Dementia Research and Education Centre, University of Tasmania, Hobart, Australia; 4 Menzies Institute for Medical Research, University of Tasmania, Hobart, Australia; 5 Human Motor Control Laboratory, School of Medicine, University of Tasmania, Hobart, Australia; 6 Research Institute for Sport and Exercise Sciences, Liverpool John Moores University, Liverpool, United Kingdom

## Abstract

Repetitive transcranial magnetic stimulation (rTMS) is commonly used to modulate cortical plasticity in clinical and non-clinical populations. Clinically, rTMS is delivered to targeted regions of the cortex at high intensities (>1 T). We have previously shown that even at low intensities, rTMS induces structural and molecular plasticity in the rodent cortex. To determine whether low intensity rTMS (LI-rTMS) alters behavioural performance, daily intermittent theta burst LI-rTMS (120 mT) or sham was delivered as a priming or consolidating stimulus to mice completing 10 consecutive days of skilled reaching training. Relative to sham, priming LI-rTMS (before each training session), increased skill accuracy (~9%) but did not alter the rate of learning over time. In contrast, consolidating LI-rTMS (after each training session), resulted in a small increase in the rate of learning (an additional ~1.6% each day) but did not alter the daily skill accuracy. Changes in behaviour with LI-rTMS were not accompanied with long lasting changes in brain-derived neurotrophic factor (BDNF) expression or in the expression of plasticity markers at excitatory and inhibitory synapses for either priming or consolidation groups. These results suggest that LI-rTMS can alter specific aspects of skilled motor learning in a manner dependent on the timing of intervention.

## Introduction

Repetitive transcranial magnetic stimulation (rTMS), a non-invasive form of brain stimulation is widely used to modify neural plasticity in clinical and non-clinical populations. In addition to the targeted cortical region/behaviour to be modulated, there are many rTMS parameters that can be adjusted (e.g. pulse frequency/protocol, stimulation intensity, timing of intervention when combined with behavioural training etc.) to produce different neurophysiological effects. Of the numerous rTMS protocols available, intermittent theta burst stimulation (iTBS), has gained considerable interest as it can induce long term potentiation (LTP) like plasticity in the human motor cortex^1^ with cortical excitability changes persisting up to 60 minutes after a single stimulation^2^, albeit with substantial variability between individuals^3^.

In addition to modulating cortical excitability, iTBS can be used to modify motor learning in humans^4–6^. This can be achieved by delivering rTMS as a priming stimulus (i.e. rTMS given prior to motor training)^4,6^ or as a consolidating stimulus (i.e. rTMS given after motor training)^5^. However, the stimulus intensities needed to alter motor behaviour and the potential mechanisms underlying iTBS induced neural plasticity remain unclear.

Rodent models provide a valuable adjunct to human rTMS studies, due to the powerful but invasive techniques available to investigate plasticity and its associated mechanisms (for review see Tang *et al*.)^7^. Similar to outcomes in humans, iTBS in rodents at high intensities has been shown to induce neural plasticity in the motor cortex using several outcome measures including increased motor evoked potential amplitudes^8^, changes in sensory-motor learning^9^ and in immediate early gene expression^10^. Potential mechanisms of iTBS induced plasticity in rodents come from histological and molecular studies, which suggest alterations in both excitatory and inhibitory activity^11–14^ and changes in brain derived neurotrophic factor (BDNF) expression^15^. A limitation of current rodent rTMS studies is the use of existing stimulator coils that are larger than the rodent brain, resulting in high intensity (HI; e.g. 1 T) but non-focal stimulation^16^. This offered us the opportunity to build a smaller coil that delivers more focal stimulation but at the expense of stimulation intensity^17^. We have shown that these smaller coils can induce cortical plasticity *in vivo* by potentiating the amplitude of motor evoked potentials in rats. In brain slices from the mouse motor cortex we have also recently shown that LI-rTMS with the iTBS protocol increases the excitability of layer 5 pyramidal neurons^18^, suggesting that even at low intensities iTBS can induce plasticity. Here we examined whether chronic low-intensity rTMS (LI-rTMS:120 mT) delivered at the iTBS frequency would have an effect at the network level by investigating changes to skilled motor behaviour in mice. In particular, we investigated whether delivering LI-rTMS to the motor cortex immediately prior to or after a skilled motor learning task (forelimb pellet reaching task^19^) would alter performance speed (number of reaches per minute) or performance accuracy in adult mice over 10 consecutive days of training. Furthermore, potential molecular and biochemical mechanisms underlying LI-rTMS-induced plasticity were investigated with western blot and ELISA assays to assess changes to excitatory and inhibitory post-synaptic proteins and brain derived neurotrophic factor (BDNF) at the conclusion of 10 days of motor learning.

The selected synaptic proteins were chosen as they have potential overlap between synaptic plasticity and rTMS-induced plasticity. Given the key involvement of α-amino-3-hydroxyl-5-methyl-4-isoxazolepropionic (AMPA) glutamate receptors in high intensity rTMS-induced plasticity^20,21^, we selected protein targets indicative of AMPA receptor expression, the receptor subunits GluR1 and GluR2, whose differential expression is linked to synaptic plasticity^22^. BDNF was selected given its upregulation with high and low-intensity rTMS^15,23–25^ and its involvement in motor learning^26^. As a marker of plasticity at the inhibitory synapse, we investigated gephyrin, a scaffolding protein at the post-synaptic membrane to which GABA_A_ receptors anchor^27,28^. Moreover, gephyrin expression has previously been shown to be modulated with HI-rTMS^29^.

Our results suggest that LI-rTMS can alter specific aspects of skilled motor learning in a manner dependent on the timing of intervention, but the molecular basis for these effects remains unclear.

## Results

### Accuracy of reaching

**Figure 1:**
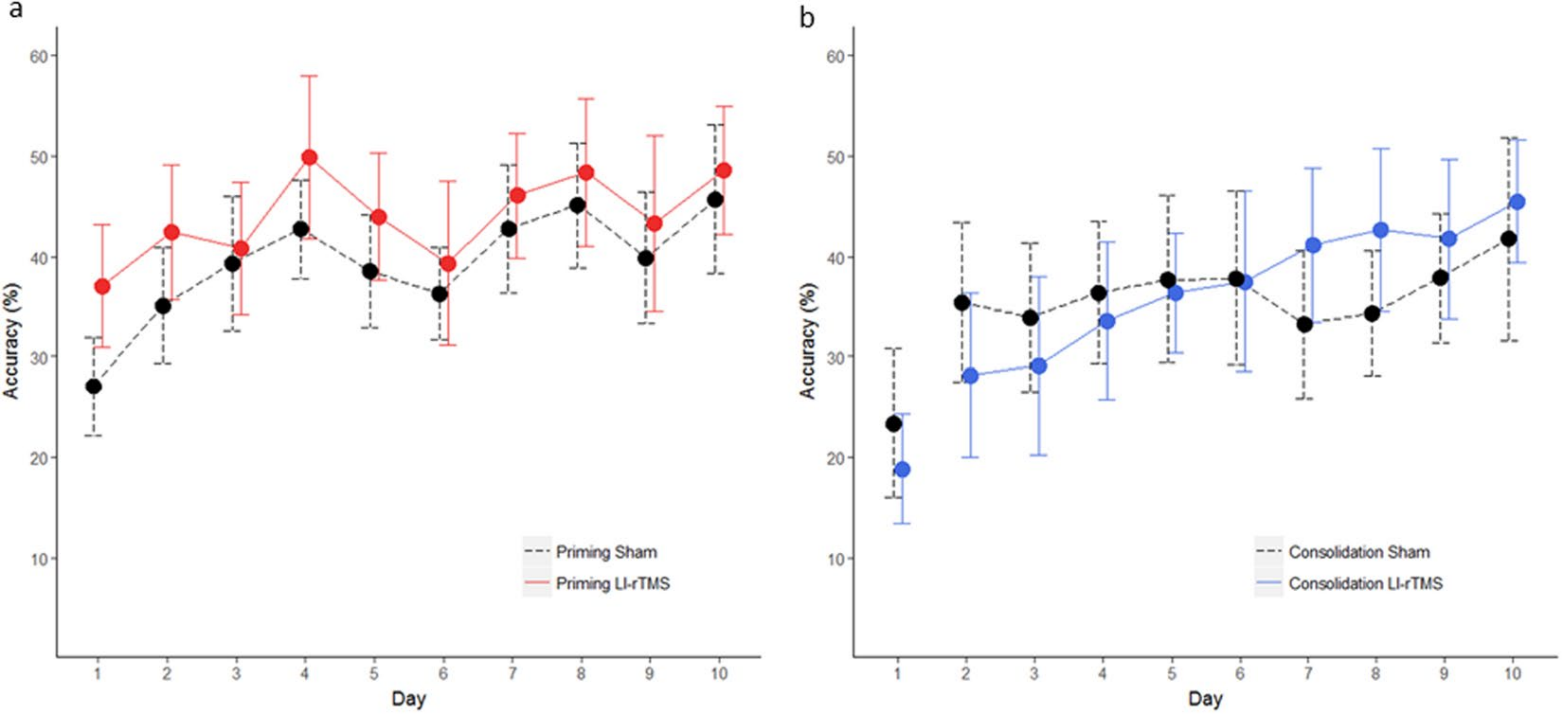
Mean reaching accuracy over 10 consecutive days with priming (n = 16 sham, n = 16 LI-rTMS) and consolidation (n = 16 sham, n = 16 LI-rTMS) stimulation. Priming LI-rTMS (**a**) increased mean skill accuracy (p = 0.02) when averaged across the whole 10 days but did not alter the rate of learning whereas consolidating LI-rTMS (**b**) increased the rate of learning (consolidating LI-rTMS*time p = 0.03) but did not alter mean skill accuracy. Only data from days 2–10 were used in the statistical model for consolidating LI-rTMS. All error bars represent 95% confidence intervals.

As expected for this task, mice (irrespective of stimulation group) became more accurate (0.6–1.4%) each day over the 10-day period (p < 0.001, see Supplementary Tables S1a and S2 for summary of statistics). Treatment with LI-rTMS significantly affected accuracy, however priming and consolidating LI-rTMS acted in different ways (Fig. 1). Priming LI-rTMS did not alter the rate of learning (priming LI-rTMS*Time interaction, p = 0.26), however mean accuracy for mice in the priming group was ~9% higher (p = 0.02) than mice in the sham group, when averaged across the entire 10 days. Taken together, a significant main effect of priming LI-rTMS and non-significant interaction with time suggests that priming LI-rTMS has an acute effect on accuracy.

In contrast, consolidating LI-rTMS altered the rate of increase in learning (consolidating LI-rTMS*Time, p = 0.03), with mice in this group becoming more accurate than sham by a further ~1.6% per day. Consolidating LI-rTMS did not significantly alter mean accuracy. Effect sizes, expressed as Cohen’s ƒ^2^ were small (ƒ^2^ = 0.04 for priming LI-rTMS, ƒ^2^ = 0.01 for consolidating LI-rTMS). (By convention, 0.02 is considered small, 0.15 is considered medium for ƒ^2^ statistics)^30^.

### Speed of reaching

Analysis of the speed of reaching showed that, mice in the priming LI-rTMS cohorts (whether sham or treatment) did not show an overall increase in speed of reaching over the time course of the experiment and that priming LI-rTMS did not alter this (see Supplementary Table S3 for priming LI-rTMS speed statistics summary). By contrast, mice in the consolidating LI-rTMS cohorts (sham or treatment) showed a slight increase in speed over time (p < 0.001, see Supplementary Table S4 for consolidating LI-rTMS speed statistics), after adjustment for weight loss (from food deprivation), with treatment acting to suppress this increase in speed relative to sham. However, the effect of consolidating LI-rTMS on speed, expressed as Cohen’s ƒ^2^, was small (ƒ^2^ = 0.027). By contrast, the effect of weight loss was ƒ^2^ = 0.112. A similar effect size differential was estimated for the priming LI-rTMS cohort, with effect sizes ƒ^2^ = 0.006 and ƒ^2^ = 0.119 for treatment and weight loss respectively.

### Molecular analysis

**Figure 2:**
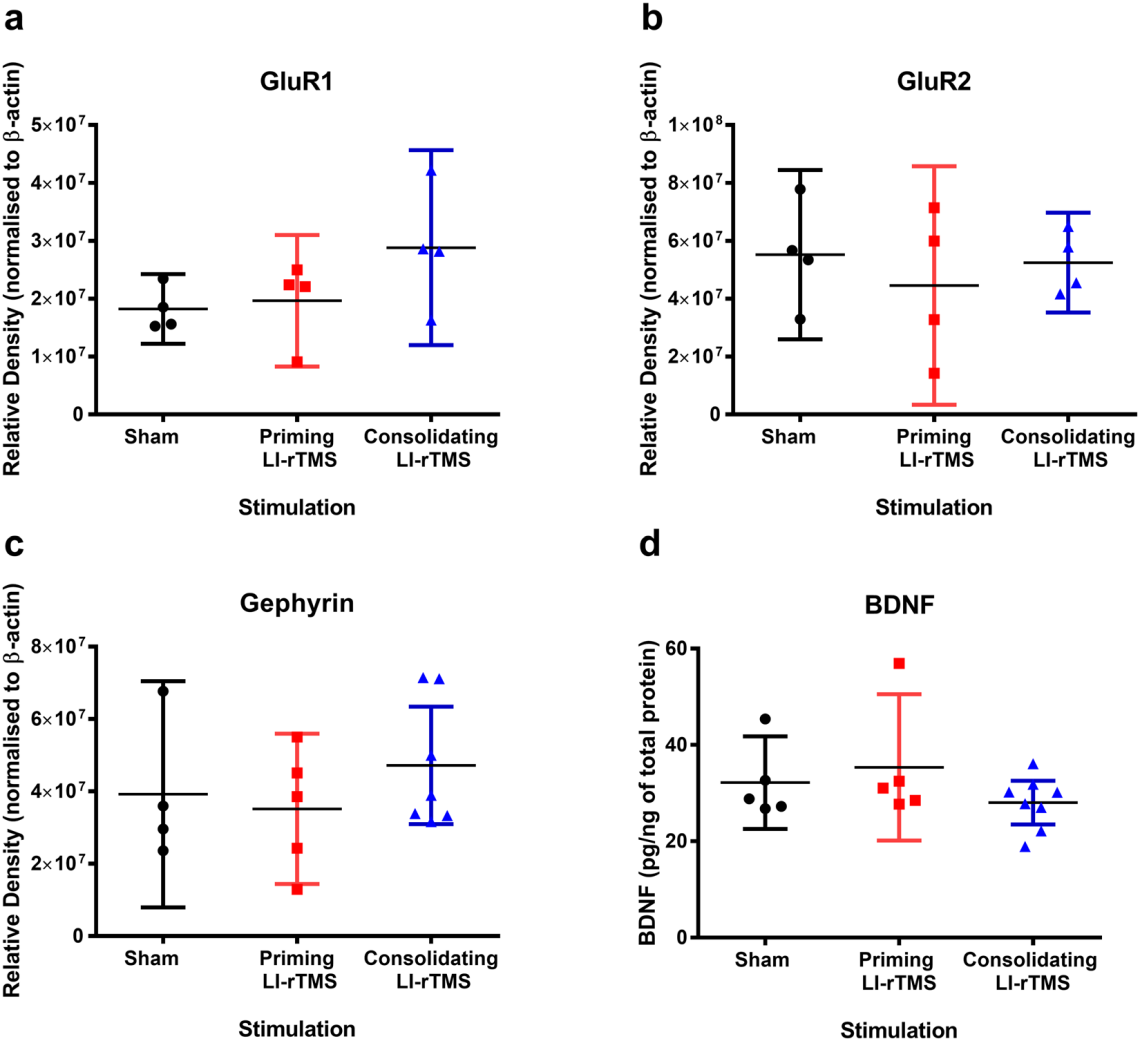
Molecular analysis of the stimulated hemisphere 24 hours post last stimulation. Semi-quantitative western blot analysis of cortical lysates showed no statistically significant differences between groups for (**a**) GluR1, (**b**) GluR2, or (**c**) Gephyrin (p > 0.05). Values on the y-axis are expressed as relative density, with all values normalised to the intensity of the β-actin loading controls. (**d**) Analysis of the BDNF ELISA showed no statistically significant differences between stimulation groups (p > 0.05). All error bars represent 95% confidence intervals.

We investigated the molecular changes persisting 24 hours after long term LI-rTMS with motor learning, using western blot and ELISA analysis on cortical tissue collected from the stimulated hemisphere, (i.e. collected 24 hours after the last stimulation on day 11) (Fig. 2).

#### Western Blots

Kruskal-Wallis analysis of western blots revealed no significant differences between groups for GluR1 (H _(2)_ = 3.73, p = 0.16), GluR2 (H _(2)_ = 0.27, p = 0.91) or gephyrin (H _(2)_ = 1.50, p = 0.50) (Fig. 2a–c).

#### Bdnf Elisa

BDNF concentrations in sham, priming and consolidating LI-rTMS animals were 32.18 ± 3.73, 35.33 ± 5.47, 28.03 ± 1.92 pg/ng of total protein respectively (Fig. 2d). A one-way ANOVA showed no statistically significant differences between the stimulation conditions F (2,18) = 1.26, p = 0.32. Follow up Dunnett’s post-hoc analysis confirmed no significant differences between sham and priming LI-rTMS (p = 0.77) or sham and consolidating LI-rTMS (p = 0.59) groups.

## Discussion

Our findings demonstrate that LI-rTMS induces behavioural plasticity which is dependent on the timing of intervention. Priming and consolidating LI-rTMS modulated specific aspects of skilled motor behaviour in adult mice. Priming LI-rTMS significantly increased skill accuracy, without altering the rate of learning (change in accuracy over time). In contrast, consolidating LI-rTMS increased the rate of learning relative to sham without altering skill accuracy. Molecular analysis of the stimulated cortical hemisphere at the conclusion of the experiments showed no change in the protein levels of AMPA receptor subunits, GABA receptor anchoring proteins and BDNF.

Our results contrast with similar priming iTBS studies in both animals and humans, in which higher rTMS intensities altered the rate of learning. Rats receiving priming iTBS at a moderate intensity (3 sessions of rTMS and training per day) learned a sensorimotor task significantly faster than sham stimulated animals^9^. Similarly, in humans, high intensity priming iTBS combined with a simple ballistic motor task resulted in an increased rate of improved performance but did not alter baseline performance^4^. Taken together these two studies suggest that moderate and high intensity priming rTMS can be used to modify the rate of motor learning but these intensities do not affect performance (i.e. skill accuracy in this study). In contrast, low-intensity priming rTMS increased performance without affecting the rate of learning.

Differences in the effect of high and low intensity rTMS on motor learning may be due to differences in the recruitment of homeostatic plasticity. In humans, four days of high intensity iTBS to the dorsal premotor cortex prior to daily mirror training resulted in significantly less skill improvement compared to controls^6^. The authors suggest that priming rTMS induces a homeostatic interaction with subsequent motor learning, such that high synaptic activity (i.e. induced with priming rTMS) increases the threshold for subsequent long term potentiation (LTP) (i.e. motor learning) and decreases the threshold for long term depression (LTD)^31^. Our results suggest that low-intensity priming rTMS has a non-homeostatic interaction with subsequent motor learning, because priming LI-rTMS increased skill accuracy but did not change the rate of learning relative to sham stimulation. Although Laapchen and colleagues stimulated the dorsal premotor cortex rather than the primary motor cortex, we suggest that the use of lower intensities may have prevented a homeostatic interaction, such that weak synaptic stimulation with priming LI-rTMS was insufficient to induce homeostatic mechanisms, but strong enough to facilitate transient increases in accuracy.

The non-homeostatic interaction of LI-iTBS with motor learning is perhaps more evident in the consolidation group, as the *rate* of motor learning was greater than sham. This finding further suggests that subthreshold intensities are strong enough to enhance LTP mechanisms but too weak to induce homeostatic interactions.

In contrast to previous studies on motor learning in mice^19^ and humans^32^, we did not see a clear-cut increase in speed over time. We found it difficult to fully account for the observed variance in speed, but notably, weight loss appeared to have a larger effect on speed than upon accuracy. Levels of hunger (satiety) therefore affect this outcome measure strongly, with mice being driven more by motivation to obtain food than by operant learning.

Despite mixed evidence^33,34^, transient enhancements in motor behaviour in humans following rTMS have been attributed to transient increases in corticospinal excitability such that motor learning may be enhanced by up-regulating the excitability of the “learning” motor cortex^35^. Although not tested in this study, we have previously investigated the acute effects of LI-rTMS on excitability within the rodent motor cortex. In acute mouse brain slices, we investigated the effect of low-intensity iTBS on neuronal excitability using single cell patch clamp recordings from layer 5 pyramidal neurons. During the 20 minutes post-stimulation (i.e. similar in time course to each motor training that proceeded priming LI-rTMS in this study), increased excitability was observed through increased evoked spike firing frequency and hyper-polarised action potential thresholds^18^. Similarly, we have shown that 10 Hz LI-rTMS to the rat motor cortex increases corticospinal excitability (increased motor evoked potential amplitudes immediately after stimulation)^17^. Therefore, combined with our previous work, the mechanisms underlying priming induced enhancements in skill accuracy may in part be due to enhanced excitability of the motor cortex (irrespective of which excitatory frequency is used) as previously suggested by human studies.

When applied as a consolidating stimulus, LI-rTMS had the opposite effect to priming, whereby consolidating LI-rTMS did not alter daily accuracy but led to an increase in the rate of motor learning over the 10 days relative to sham. Although little is known on the effect of consolidating LI-rTMS on motor behaviour, particularly over multiple sessions, our results are in contrast to the report of high intensity rTMS using iTBS, which degraded prior motor learning in humans within a single experimental session^5^. Whilst Stockel *et al*. tested motor performance immediately after the consolidating iTBS, in our study, changes in motor behaviour were not assessed until the next day (24 hours after the delivery of iTBS). Therefore, intensity aside, it is possible that as a consolidating stimulus, iTBS has different effects on early (e.g. within 30 minutes of stimulation) and later aspects of motor consolidation, such as sleep-dependent and structural mechanisms. In mice, repeated skilled pellet reaching leads to complex structural remodelling of dendritic spines^36,37^, such that there is an increase in the number of new dendritic spines formed (spinogenesis) and a destabilisation of pre-existing spines during motor learning^36^. Therefore, our increase in motor learning following low-intensity consolidating iTBS may be due to changes in structural plasticity, as shown *in vitro*^20^ and will require further investigation.

Interestingly, molecular analysis of the stimulated cortical hemisphere with priming and consolidating LI-rTMS revealed no long lasting changes to key synaptic plasticity markers or BDNF. It is possible that we did not detect changes in protein expression because our methods were based on homogenised tissue of the stimulated hemisphere and did not examine changes in protein localised to specific brain regions (i.e. purely motor cortex), neuronal subtypes, or even subcellular localisation. The importance of subcellular protein trafficking in mediating rTMS effects has been recently shown by altered distribution of gephyrin protein clusters, in hippocampal cultures following 10 Hz repetitive magnetic stimulation (rMS)^29^. Interestingly, changes in gephyrin cluster size and number were not accompanied by changes in the amount of gephyrin mRNA or protein expression^29^. Therefore, the plasticity induced by LI-rTMS in our study may have been underpinned by mechanisms similar to those known to occur following 10 Hz rMS *in vitro*, involving changes in protein trafficking and distribution rather than changes in the amount of expression.

Alternatively, as our analysis was conducted on tissue collected 24 hours after the last stimulation (i.e. day 11), we cannot rule out the possibility that changes in molecular markers occurred between days 1 and 10 but were not sustained to day 11. Similarly, stimulation may have induced transient changes (e.g. <24 hours post-stimulation) which we could not detect at the time point investigated. This is supported by the evidence that a single session of rMS in organotypic slice cultures induces structural and functional plasticity that occur ~2 hours post-stimulation but are absent at 6 hours post stimulation^20^.

To our knowledge, this is the first study to investigate the effect of chronic low-intensity iTBS on skilled motor behaviour. Compared to previous studies that use commercial human rTMS coils in laboratory animals, the use of a custom rodent-specific coil allowed for preferential (more focal) stimulation of the motor cortex^17^ contralateral to the dominant forepaw and avoided direct stimulation of the ipsilateral motor cortex and other motor regions such as the cerebellum, known to influence motor learning in humans^38^ and rodents^39^. However, it is possible that cortical regions adjacent to the targeted motor cortex may have been stimulated. In particular, stimulation of the dorsal anterior cingulate cortex (adjacent to the secondary motor cortex), implicated in attention control^40^, may have contributed to the observed effects on motor-skill, particularly in the priming LI-rTMS animals (i.e. LI-rTMS primed animals may have been more attentive to the presence and replacement of the food pellets). Another limitation of our small coils is the inability to stimulate at high intensities due to high mechanical instability and heat generation^41^. Therefore, although a direct comparison of low and high intensity rTMS is of great interest, focal high intensity rTMS in mice will require other tools, such as intracortical electrical stimulation as a proxy for rTMS^42^. Alternatively, studies may be carried out in rats, where a degree of focality can be achieved with commercial coils^8,43^.

Whilst our results suggest that LI-rTMS may be more beneficial for chronic and repeated stimulation in the intact nervous system to avoid homeostatic interactions, the effect sizes observed were modest (e.g. ~9% greater increase in accuracy with priming rTMS). Therefore, it is unclear whether LI-rTMS offers meaningful benefit to the intact motor system. However, it is worth noting that in line with our results, intact wild-type mice typically show a maximum increase in accuracy of 15% over the training period in the single pellet reaching task^36,44^. Future studies employing skilled motor tasks that allow for a larger range of improvement (i.e. >20%) or in combination with other skilled motor tasks (e.g. rotarod) will be necessary to determine the potential of LI-rTMS in the intact motor system.

Similarly, the use of LI-rTMS for the treatment of neurotrauma and neurological disorders where cortical plasticity may be abnormal, may have greater benefit than in the intact nervous system. For example, rats with stroke infarcts of the forelimb area were ~20% more accurate in pellet retrieval after combined intracortical stimulation of the motor cortex and training than training only animals^45^. Therefore, future studies investigating LI-rTMS on functional outcomes in pre-clinical injury and disease models would reveal which populations and disorders might benefit from LI-rTMS.

## Materials and Methods

### Animals

10 week old male C57Bl/6 J mice (*Mus Musculus*) (UTAS colony originally derived from Jackson Laboratory stock) were group housed on a 12-hour light/dark cycle. Two days prior to the commencement of behavioural testing, animals began food restriction (~2 hours of food:Barastoc irradiated mouse cubes, 6% fat content per day, given each afternoon after the end of behavioural testing) to reduce and maintain animals at ~90% of baseline weights throughout the experiment (animal weights were recorded daily prior to behavioural testing and factored into the analysis as weight loss). Water was provided *ad libitum*. 24 hours after the end of experimentation, mice were euthanized with an overdose of pentabarbitone (>160 mg/kg i.p injection). All animal experimentation was performed in accordance with the Australian code of practice for the care and use of animals for scientific purposes and was approved by the University of Tasmania Animal Ethics Committee (A0013168).

### Custom rodent rTMS coil

A custom rodent-specific circular coil (8 mm height × 8 mm outer diameter, steel core, 780 turns of 0.125 mm diameter wire) was used to deliver more focal rTMS and avoid direct stimulation of other cortical regions involved in motor function (e.g. cerebellum) that might occur with larger commercial coils (for further coil specifications see Tang *et al*.)^17^. Monophasic stimulation pulses (400 µs rise time) were delivered by a waveform generator (Agilent 335141B, USA) connected to a bipolar voltage programmable power supply (KEPCO BOP 100-4 M, USA). Experiments were conducted at 100% of the maximum power supply output (±100 V) (Agilent Benchlink Waveform Builder, USA). Peak magnetic field strength was 120 mT at the surface of the coil (measured with a Hall-effect probe Honeywell SS94A2D, USA). Maximum stimulation at the base of the coil equated to a dB/dT of 400 T/s.

### Repetitive Transcranial Magnetic Stimulation

iTBS patterned LI-rTMS or sham stimulation was delivered daily for 10 consecutive days to the motor cortex contralateral to the dominant/trained forelimb of lightly restrained awake mice, by placing the mouse in a restraint bag with a breathing hole at one end (Able Scientific). The TMS coil was positioned over the mouse cranium, such that the coil windings overlaid the dominant motor cortex, with the coil offset laterally to minimise direct stimulation of the non-dominant motor cortex. During stimulation, the coil was held ~1 mm over the mouse cranium. Six hundred pulses of iTBS (pulse trains consisting of 3 pulses at 50 Hz, repeated at 5 Hz and delivered for 2s, with an inter-train interval of 8s)^1^, lasting 190 seconds was delivered once daily for 10 consecutive days, either immediately before or after motor training. Sham stimulation consisted of manual restraint for 190s as described above.

### Skilled Motor Training: Single-Pellet Reaching Task

The single pellet-reaching task is a skilled forelimb behavioural task that is analogous to throwing darts or shooting basketballs in humans^46^. Briefly, a single animal was placed into a clear perspex chamber (20 cm tall, 15 cm deep and 8 cm wide). A vertical slit (10 mm wide) in the centre of the chamber allowed the animals to retrieve a food reward (2.5 mm diameter, 20 mg chocolate flavour grain pellet, Bio-Serv, New Jersey) placed on a 10 mm high platform in front of the slit. Pellets were replaced by the experimenter immediately after (i) the pellet was grasped by the animal or (ii) the pellet was displaced following the previous reaching attempt.

Animals underwent pre-training (“shaping”) once a day until a baseline level (20 reaches made within a 20-minute training session) was achieved. In addition, the animals’ individual learning abilities were defined based on the number of shaping days needed until baseline level was achieved (“fast learner” = 1 day of shaping required, “intermediate learner” = 2 to 3 shaping days required or “slow learner” = 4 shaping days required). Animals that did not reach baseline level after 4 days shaping were excluded from further testing. For shaping, pellets were placed in the centre of the slit, allowing the animal to reach with both forelimbs. The dominant forelimb was defined as the forelimb used in >70% of reaching attempts. Groups were counterbalanced such that an equal number of learning abilities (shaping speed and paw dominance) were placed into sham and treatment groups. Groups were also balanced by litter wherever possible, with equal numbers from each box of group-housed males in sham and treatment groups. After stratification by shaping speed, paw dominance and litter, mice were randomly allocated to treatment groups. Four cohorts of animals were tested in separate experiments (two cohorts receiving priming LI-rTMS or sham, two receiving consolidating LI-rTMS or sham). In total, priming LI-rTMS n = 16, consolidating LI-rTMS n = 16 and n = 16 sham animals were tested in each of these two experimental groups

**Figure 3:**
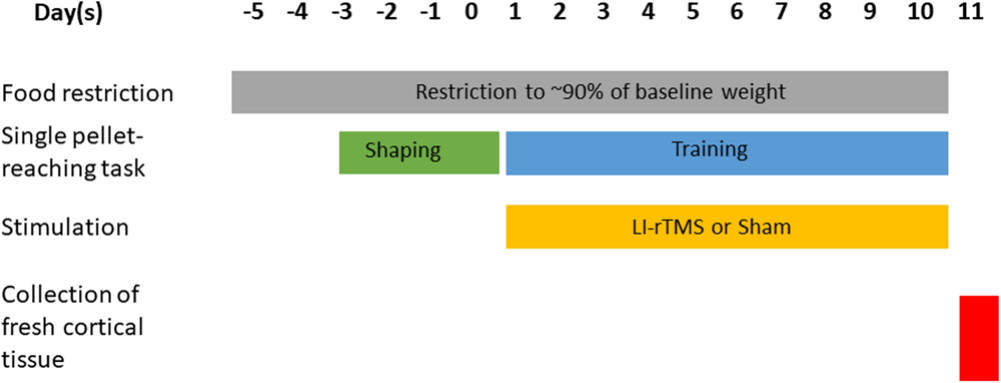
Outline of experimental paradigm. Animals were food restricted to ~90% of baseline weight throughout the experiment. Following 4 days of shaping (pre-training), animals were allocated into stimulation groups (sham or LI-rTMS) followed by 10 consecutive days of skilled pellet-reaching training. LI-rTMS or Sham stimulation was given immediately before (priming) or after (consolidation) each training session. 24 hours after the last stimulation, cortical tissue from the stimulated/dominant hemisphere was collected for molecular analysis of select neuroplasticity markers.

Once shaped, animals underwent 10 consecutive days of motor training, with each daily session consisting of 30 reaches or 20 minutes of training, whichever was achieved first (see Fig. 3 for outline of experimental paradigm). Animals received one session of motor training and stimulation at the same time each day for 10 consecutive days and each training session was recorded with a digital camcorder to verify online scores with offline analysis. The pellet was placed to one side of the slit midline such that the animal could only collect it by reaching with its pre-determined dominant forelimb (preventing reaching with non-dominant hand and tongue). Reaching attempts were scored as a success (dominant forelimb grasped and retrieved pellet and then placed it into the mouth), a drop (dominant forelimb grasped and retrieved pellet but dropped it before placing it into the mouth) or a fail (an attempt with the dominant forelimb that missed or misplaced the pellet). Drops and fails were grouped together and classified as fails overall.

### Molecular Biology Tissue preparation

Twenty-four hours after the last period in the testing chamber, mice were terminally anaesthetised with 160 mg/kg pentabarbitone sodium. Brains were rapidly removed and dissected for hippocampus or overlying cortex (a region including both motor and sensory cortex). Samples were taken from the dominant hemisphere (i.e. the hemisphere that received stimulation). Samples were snap frozen with liquid nitrogen and stored at −80 °C.

### Western blotting

Dominant hemisphere samples were homogenised in 1 mL of ice-cold lysis buffer designed to maximise yield of BDNF^47^ (100 mM PIPES pH7, 500 mM NaCl, 0.2% Triton X-100, 2 mM EDTA) containing EDTA-free protease inhibitor cocktail (Roche Biochemicals). Homogenised lysates were sonicated then centrifuged (3320 × g at 4 °C for 1 hour) and supernatants stored at −80 °C. Protein levels were quantitated via the bicinchoninic acid assay as per manufacturer’s instructions (Thermo Fisher) and equal amounts (approx. 10 µg) loaded onto 10% or 12% Tris-glycine reducing SDS-PAGE gels. Gels from all 3 groups were transferred onto a single PVDF membrane (Millipore) for each synaptic marker in an interleaved fashion, so that each group was evenly distributed across the entire width of the membrane to minimise intra-group errors due to localised differences in staining. Membranes were blocked with PBS + 0.1%Tween containing 5% skimmed milk powder and probed with primary antibodies for synaptic markers and beta-actin simultaneously as follows: GluR1 (Abcam ab31232); GluR2 (Neuromab L:21/3275-002); Gephyrin (Abcam ab25784); β-actin (Sigma A2228). Membranes were incubated with peroxidase-conjugated secondary antibodies to rabbit or mouse (Dako) and imaged with chemiluminescent substrate (Millipore) using an Amersham Imager 600 (GE Healthcare) then quantitated using ImageJ, with targets of interest normalised to β-actin.

### Bdnf Elisa

Cortical lysates were assayed using a commercial sandwich ELISA kit as per manufacturer’s instructions (Chemikine, Millipore) and all samples run in triplicate. BDNF levels were normalised to total protein concentration.

### Data Analysis and Statistics

Behavioural videos for each training session were analysed for two outcome measures of skilled motor learning: accuracy and speed. Accuracy was defined as the percentage of successful reaches relative to the total number of reaches made. Speed was defined as the total number of reaches made per minute, regardless of success. Rate of learning was defined as the change in accuracy across sessions.

Linear mixed effects models were used to estimate the effect of Ll-rTMS treatments on pellet reaching tasks, using the *nlme* package v3.1-131^48^ and *lme4* package v1.1-13^49^ in R v3.4.1 (R Core Team, 2017). Mixed-effect models are more appropriate than repeated measures ANOVA for these data sets because they allow for missing data, the estimation of multiple random effects and the treatment of time as a continuous covariate. The initial full model included: a random slope within-subject, to take into account variability in motor learning for individual mice; a random intercept for each animal, to account for individual differences in baseline performance levels and learning ability; and a random intercept for cohort, to model potential differences between groups of animals (experiments) caused by uncontrolled external factors (e.g. time of year and ambient temperatures when experiments were performed). Model selection proceeded using a top-down approach^50^ and the initial full models specified fixed effects for time (day), stimulation (as a two-level factor for sham and either priming LI-rTMS or consolidating LI-rTMS), weight loss (%) and shaping speed (as a three-level factor for fast/intermediate/slow learners) and included a term for interaction between time and stimulation. Preliminary model fitting used Akaike’s Information Criterion (AIC) and F-tests on models fitted using Restricted Estimation of Maximum Likelihood (REML) to estimate informative random effects. An unstructured covariance matrix was used to model the random effects. Fixed effects were then selected using Likelihood Ratio Tests (LRT), comparing the model with the fixed effect of interest against a model without it, fitting each model using the Maximum Likelihood (ML) method. The speed variable (reaches/minute) was log transformed. Residual diagnostics were assessed using standard graphical methods for linear models. Observations for day 1 were stripped from the dataset for the consolidation experiment, as these observations *preceded* treatment. These models therefore only include data from days 2 to 10.

A single, parsimonious model was specified to explain accuracy and speed for both priming and consolidation treatments,

$${\rm{Y}}={\rm{weight}}\_{\rm{loss}}+{\rm{treatment}}+{\rm{day}}+{\rm{treatment}}\ast {\rm{day}}+({\rm{day}}|{\rm{id}})+(1|{\rm{cohort}})$$

where weight loss is expressed as a percentage, treatment is a factor with two levels denoting treatment with LI-rTMS or sham treatment and the terms in brackets specify the random effects structure in lme4, with a random slope and intercept for each animal and a random intercept for each cohort^51^.

Type III F-tests were computed using the Kenward-Roger approximation for estimating degrees of freedom and the F statistic. Cohen’s ƒ^2^ statistics for standardized local effect size (ƒ^2^ = (*R*^2^_*AB*_ − *R*^2^_*A*_*)/(1* − *R*^2^_*AB*_)) *were* calculated using marginal *R*^2^ coefficients following the method described by Nakagawa & Schielzeth^30^. Post-hoc tests to examine mean differences between sham and LI-rTMS groups on each day was *not* conducted as they do not account for multiple random effects or weight loss, which would result in greater likelihood of type 1 error.

To analyse the western blot intensities (normalised to β-actin), Kruskal-Wallis tests were performed.

The datasets generated during and/or analysed during the current study are available from the corresponding authors on reasonable request.

## Electronic supplementary material

Supplementary Material
