# Supplementary material for: Low intensity repetitive transcranial magnetic stimulation modulates skilled motor learning in adult mice
Source: Sci Rep. 2018 Mar 5;8:4016. doi: 10.1038/s41598-018-22385-8 (PMC5838100; doi:10.1038/s41598-018-22385-8)
Supplement: Supplementary file 1 — Supplementary Material [file 41598_2018_22385_MOESM1_ESM.pdf]

Low intensity repetitive transcranial magnetic stimulation modulates skilled motor learning in adult mice

Alexander D Tang [1,2+*], William Bennett[3+*], Claire Hadrill[3], Jessica Collins[3], Barbora Fulopova[3], Karen Wills[4], Aidan Bindoff[3], Rohan Puri[5], Michael I Garry[5], Mark R Hinder[5], Jeffery J Summers[5,6], Jennifer Rodger[1] and Alison J Canty[3].

1. Experimental and Regenerative Neurosciences, School of Animal Biology, University of Western Australia, Perth, Australia.
2. Neuronal Rhythms in Movement Unit, Okinawa Institute of Science and Technology Graduate University, Okinawa, Japan.
3. Wicking Dementia Research and Education Centre, University of Tasmania, Hobart, Australia.
4. Menzies Institute for Medical Research, University of Tasmania, Hobart, Australia.
5. Human Motor Control Laboratory, School of Medicine, University of Tasmania, Hobart, Australia.
6. Research Institute for Sport and Exercise Sciences, Liverpool John Moores University, Liverpool, United Kingdom.

[+] These authors contributed equally to this work

Supplementary Material

Supplementary Table S1 – Priming LI-rTMS accuracy statistics summary table

Table 1a - Modelling the effect of time and/or priming LI-rTMS treatment upon accuracy of reaching<br>(coefficent values are expressed as percentage of accurate reaches)			
* denotes significance at $p<0.05$ level<br>** denotes significance at $p<0.01$ level<br>*** denotes significance at $p<0.001$ level			
**fixed effect**	**coefficient (β)**	**standard error (s.e.m.)**	**p value**
intercept	28.09	3.00	0.00
time (day)	+1.38	0.38	0.001***
priming LI-rTMS	+8.67	3.51	0.02*
interaction - priming LI-rTMS * time	-0.64	0.55	0.257
weight loss	+0.34	0.16	0.041*

Supplementary Table S2 – Consolidation LI-rTMS accuracy statistics summary table.

Table 1b - Modelling the effect of time and/or consolidating LI-rTMS treatment upon accuracy of reaching<br><br>(coefficient values are expressed as percentage of accurate reaches)			
* denotes significance at p<0.05 level<br><br>** denotes significance at p<0.01 level<br><br>*** denotes significance at p<0.001 level			
**fixed effect**	**coefficient (β)**	**standard error (s.e.m.)**	**p value**
intercept	34.65	9.45	0.09
time (day)	+0.55	0.49	0.001***
consolidating LI-rTMS	-8.97	4.90	0.08
interaction - consolidating LI-rTMS * time	+1.59	0.69	0.03*
weight loss	-0.15	0.32	0.65

Supplementary Table S3 – Priming LI-rTMS speed statistics summary table

Table 2a - Modelling the effect of time and/or priming LI-rTMS treatment upon speed of reaching expressed as $\log_e$ (reaches/minute).			
* denotes significance at p<0.05 level<br>** denotes significance at p<0.01 level<br>*** denotes significance at p<0.001 level			
**fixed effect**	**coefficient (β)**	**standard error (s.e.m.)**	**p value**
intercept	0.05	0.18	0.81
time (day)	+0.03	0.02	0.131
priming LI-rTMS	0.109	0.14	0.438
interaction - priming LI-rTMS * time	-0.03	0.02	0.269
weight loss	+0.04	0.01	<0.001***

Supplementary Table S4 – Consolidation LI-rTMS speed statistics summary table

Table 2b - Modelling the effect of time and/or consolidating LI-rTMS treatment upon speed of reaching expressed as $\log_e$ (reaches/minute).			
* denotes significance at p<0.05 level<br>** denotes significance at p<0.01 level<br>*** denotes significance at p<0.001 level			
**fixed effect**	**coefficient (β)**	**standard error (s.e.m.)**	**p value**
intercept	-0.11	0.45	0.84
time (day)	+0.09	0.02	<0.001***
consolidating LI-rTMS	+0.23	0.20	0.27
interaction - consolidating LI-rTMS * time	-0.07	0.02	0.01*
weight loss	+0.09	0.01	<0.001***
